# Electrospun Silver Nanoparticles-Embedded Feather Keratin/Poly(vinyl alcohol)/Poly(ethylene oxide) Antibacterial Composite Nanofibers

**DOI:** 10.3390/polym12020305

**Published:** 2020-02-03

**Authors:** Ming He, Man Chen, Yao Dou, Jiao Ding, Hangbo Yue, Guoqiang Yin, Xunjun Chen, Yingde Cui

**Affiliations:** 1 College of Chemistry and Chemical Engineering, Zhongkai University of Agriculture and Engineering, Guangzhou 510225, China; cm1601616081@163.com (M.C.); chj.ding@163.com (J.D.); cxj.qiao@163.com (X.C.); 2 Innovation and Practice Base for Postdoctors, Chengdu Polytechnic, Chengdu 610041, China; douyao1019@aliyun.com; 3 School of Chemical Engineering & Light Industry, Guangdong University of Technology, Guangzhou 510006, China; hangbo.yue@gdut.edu.cn; 4 Guangzhou Vocational and Technical University of Science and Technology, Guangzhou 510550, China; 13602880087@139.com

**Keywords:** keratin, silver nanoparticles, electrospinning, nanofibers, antibacterial activity

## Abstract

Feathers, which contain >90% keratin, are valuable natural protein resources. The aim of this study is to prepare antimicrobial feather keratin (FK)-based nanofibers by incorporating silver nanoparticles (AgNPs). A series of AgNPs-embedded feather keratin/poly(vinyl alcohol)/poly(ethylene oxide) (FK/PVA/PEO) composite nanofibers with varying amounts of AgNPs content were fabricated by electrospinning. Their morphology, crystallinity, thermal stability, tensile property, and antibacterial activity were systematically investigated. The average diameters of composite nanofibers gradually decreased with increases in the amount of AgNPs. The crystallinity, thermal stability, and antibacterial activity of FK/PVA/PEO nanofibers were enhanced by embedding AgNPs. When embedded with 1.2% AgNPs, both the tensile strength and elongation-at-break reached the highest level. This work has the potential to expand the application of FK-based nanofibers in the biomaterial field.

## 1. Introduction

In the past two decades, electrospun nanofibers have gained considerable attention because of their special structural characteristics and functional properties. They are extensively used for wound dressings, drug delivery, tissue engineering, heavy metal ion adsorption, and active packaging of antimicrobials [1,2,3,4,5]. Recently, researchers are increasingly focused on the combination of natural polymers and synthetic polymers to produce composite nanofibers using an electrospinning process. For these composite nanofibers, strong mechanical strength, preferable biocompatibility, and biodegradability are expected to be simultaneously obtained. On the other hand, although solution electrospinning is fast developed to produce complex nanostructures through 2-fluid coaxial [6,7], 3-fluid tri-axial [8], and side-by-side electrospinning process [9], the treatment of new type of materials into nanofibers using a single-fluid electrospinning process is always highly desired because of the limited filament-forming polymer matrices.

Poly(vinyl alcohol) (PVA) and poly(ethylene oxide) (PEO) are hydrophilic polymers with excellent biocompatibility, mechanical performance, and low toxicity. Moreover, both PVA and PEO are easily electrospinnable because of their high molecular weight and viscosity. Thus, there have been many studies on the composite nanofibers of PVA- and PEO-blended with keratin [10,11], soy protein [12,13], whey protein isolates [14,15], alginates [16,17], and chitosan [18,19,20]. Importantly, blending modification with PVA or PEO can significantly facilitate the electrospinning process for polymers that do not have sufficient entanglement or interactions for electrospinning [21].

Keratin is considered as one of the most abundant proteins that can be obtained from hair, wool, feathers, as well as horns of reptiles, mammals, and birds [22]. To date, keratin has been extensively utilized to prepare nanofibers in various biomedical applications because of its biocompatibility and biodegradability [23,24]. Wu et al. [25] found that adding keratin to the PCL nanofiber can improve the hydrophilicity of the as-spun nanofiber mats and then promote the cell adhesion and proliferation of the composite nanofibrous mats. Moreover, the membranes showed faster degradation with increasing of keratin content. The porosity swelling by the biodegradation of keratin and collapse of composites provided highly interconnected porous networks to facilitate cell migration, nutrient delivery, and waste exchange, resulting in high cell viability. Feathers, which contain >90% keratin, are byproducts of the poultry industry. A number of feathers are discarded as solid waste in landfills every year [26]; moreover, discarded feathers have caused environmental pollution and natural protein resource waste. Therefore, the recycling of feather keratin (FK) is attracting considerable attention from researchers.

The application of nanofibers in the biomedical field requires antimicrobial properties for materials. One approach is incorporating inorganic nanoparticles, among which silver nanoparticles (AgNPs) played an important alternative antibacterial agent and have been studied against many types of microbes [27]. Because of their antimicrobial activities, AgNPs have been extensively used for preparing antimicrobial nanofibers. Aktürk et al. [28] added starch-coated AgNPs into PVA solutions and successfully incorporated AgNPs in the PVA matrix to fabricate nanofibers using an electrospinning process. Moreover, they determined that nanofibers containing up to 10 (wt/wt)% S-AgNPs content could maintain a porous and nanofibrous structure. Antibacterial assays of S-AgNPs-incorporated PVA nanocomposite mats showed that clearer and larger circular zone inhibition was observed against *Staphylococcus aureus* compared to *Escherichia coli*. Tra Thanh et al. [29] reported the preparation that PCL nanofiber membranes were coated using AgNPs embedded in gelatin (Gel) by multi-immersing the membranes into Gel-Ag solutions. Moreover, the antibacterial effect of multi-coated membranes was more significant compared to the single coating one. In addition, the incorporation of AgNPs acted as conductive nanofillers in the polymer matrix improves conductivity of the spinning solution. Thus, more charge density in the solution increases the columbic forces and stretching of the solution during the electrospinning process, resulting in the preparation of nanofibers with smaller diameter [30,31].

The electrospinning of FK/PVA two-component and FK/PVA/PEO three-component nanofibers have been reported in our previous studies [32,33]. The aim of the current study was intended to improve the antimicrobial properties of FK/PVA/PEO nanofibers by embedding AgNPs. Water-dispersible and surface-modified AgNPs were synthesized using liquid phase reduction with PVP acting as a surface modification agent. The influence of incorporated AgNPs content on the morphologies and properties of FK/PVA/PEO composite nanofibers was systematically investigated.

## 2. Materials and Methods

### 2.1. Materials

The feather keratin (FK) powders were extracted from chicken feathers, using the method described in our previous study [33]. PVA (degree of alcoholysis: 87–89%) and PEO (Mw = 400,000) were purchased from Aladdin Industrial Corporation (China). Other agents used were of analytical grade and all solutions were prepared using distilled water.

### 2.2. Synthesis of Water—Dispersible AgNPs

Water-dispersible AgNPs were prepared using the method reported by Zhang with some modification [34]. PVP was previously dissolved with distilled water in a 250 mL flask. Tannic acid (2.4 mmol/L) and NH_3_·H_2_O (0.5 mol/L) were added to the flask with mechanical stirring at room temperature, and then the color of the solution turned brown. Then, AgNO_3_ (120 mmol/L) was dropwise injected into the flask, and the reaction solution gradually turned black. The reaction was allowed to proceed for 1 h, and the resulting solution was concentrated for 4 h using a rotary evaporator at 60 °C. Subsequently, acetone was added to the concentrated solution. The precipitate was then filtered and cross-washed with distilled water and acetone several times. Finally, the water-dispersible AgNPs were obtained via vacuum drying at room temperature.

### 2.3. Preparation of Electrospinning Solutions

The electrospinning solution comprised the three-component polymer solution and AgNPs solution. The FK solution (12 wt%) was prepared by thoroughly dissolving the as-prepared FK powders in distilled water with 3 mol·L−1 NaOH solution slowly dropping with continuous stirring at 50 °C. PVA (12 wt%) and PEO (12 wt%) solutions were prepared by dissolving the required amount of corresponding powders in distilled water with continuous stirring at 80 °C. The three-component polymer solution was obtained by mixing FK solution (3 g), PVA solution (8.4 g), and PEO solution (3.6 g). AgNPs solution (0.5% wt%) was prepared by dispersing the as-synthesized AgNPs in distilled water. To obtain the electrospinning solution, the AgNPs solution with different contents was added to the three-component polymer solution. A series of spinning solutions was coded as m-AgNPs, where m was the weight percentage of AgNPs to the total three-component polymers.

### 2.4. Electrospinning of AgNPs-Embedded FK/PVA/PEO Composite Nanofibers

Antibacterial composite nanofibers were prepared using an electrospinning machine (ET-2535DC, Beijing Yongkang Leye Technology Development Co. Ltd., Beijing, China). Each spinning solution (10 mL) was loaded into a syringe equipped with a 22-gauge blunt-tipped needle. A rotating cylindrical mandrel wrapped with an aluminum foil was used as a fiber collector. The electrospinning process was performed at an applied voltage of 20 kV, a needle tip-to-collector distance of 15 cm, and a flow rate of 0.6 mL/h.

### 2.5. Characterization

The morphology and microstructure of electrospun composite nanofibers were observed by a transmission electron microscope (TEM, JEM-2010HR, JEOL, Tokyo, Japan) and a scanning electron microscope (SEM, EVO 18, Carl Zeiss, Jena, Germany) equipped with an EDX analyzer. The average diameter and diameter distribution of nanofibers were determined by measuring 100 fibers that were randomly selected from each sample.

The crystallinity of the sample was determined by X-ray diffraction (XRD, Empyrean, PANalytical Company Ltd., Almelo, Netherlands) with Cu Kα irradiation at an applied voltage of 40 kV, a 2θ scan range of 5–90°.

Thermal stabilities were assessed by thermogravimetric analysis (TGA, Instruments TG209F1, Netzsch, Germany) in the temperature range of 40−700 °C with a heating rate of 10 °C/min, and samples of 8–10 mg were used.

The tensile properties of composite nanofibers, including tensile strength (TS) and elongation-at-break (EAB), were determined using a microcomputer-controlled electronic universal testing machine (CMT6503, Shenzhen MTS Test Machine Company Ltd., Shenzhen, China). According to the ASTM standard D638, a strain rate of 10 mm/min and a fixture distance of 40 mm were used throughout the experiment. The nanofibers were cut into samples measuring 75 × 10 mm, and the thicknesses of the samples were measured by a micrometer. Three replications of each sample were obtained, and the average values of the measurements were used.

### 2.6. Antibacterial Assays

The antibacterial assays of the AgNPs-embedded FK/PVA/PEO composite nanofibers were tested against *Escherichia coli* (CICC 10003, Gram-negative) and *Staphylococcus aureus* (CICC 10001, Gram-positive) using the agar-diffusion method. The nanofiber samples were cut into a disc shape with 10 mm diameter and heated for 6 h at 150 °C to improve the resistance to water. Then the self-crosslinking disc samples were sterilized under UV irradiation for 2 h (each side for 1 h), and 100 μL of bacterial suspension (*E. coli* or *S. aureus*) was uniformly spread on Luria–Bertani (LB) agar plates using sterile glass spreaders. The sterilized disc samples were placed on the agar plates, and then incubated at 37 °C for 24 h in an incubator. The antibacterial activities of the composite nanofibers were quantified by evaluating the diameter of the inhibition zone around each disc, which showed the efficacy of the samples against different bacterial species.

## 3. Results

### 3.1. Appearance and Microstructure

**Figure 1:**
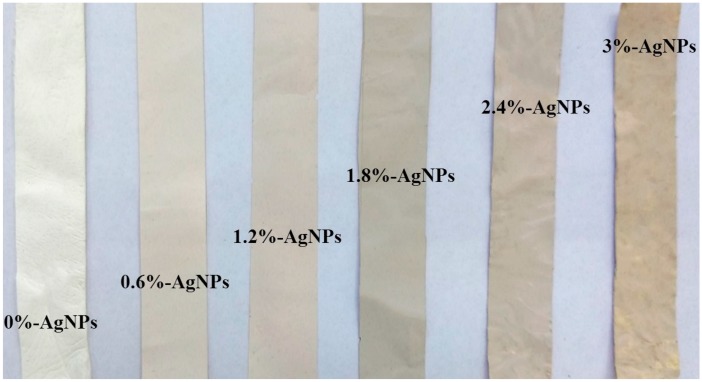
Photos of feather keratin/poly(vinyl alcohol)/poly(ethylene oxide) (FK/PVA/PEO) composite nanofibers with different AgNPs content.

**Figure 2:**
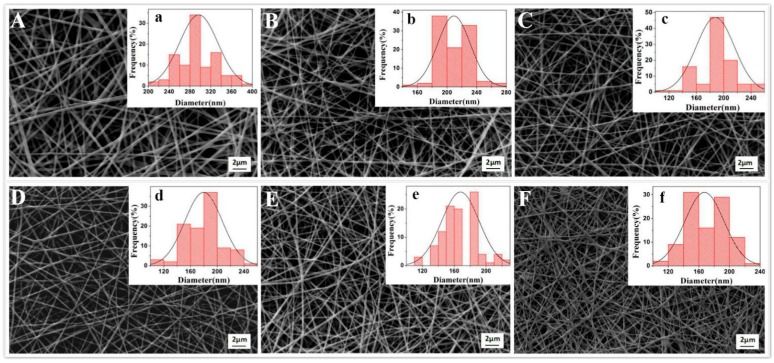
SEM images and diameter distributions of FK/PVA/PEO composite nanofibers with different AgNPs contents: (**A**,**a**) 0%-AgNPs, (**B**,**b**) 0.6%-AgNPs, (**C**,**c**) 1.2%-AgNPs,(**D**,**d**) 1.8%-AgNPs, (**E**,**e**) 2.4%-AgNPs, (**F**,**f**) 3%-AgNPs.

At the fixed spinning conditions, the electrospinning process of AgNPs-induced spinning solutions was stable and successful for all experimental groups. As shown in Figure 1, the FK/PVA/PEO composite nanofibers became darker with increase in AgNPs content. SEM and TEM observations were performed to obtain a better insight in the microstructure of composite nanofibers. The SEM images, diameter distributions, and average diameters of the composite nanofibers with different AgNPs contents are shown in Figure 2 and Table 1. The 0%-AgNPs nanofibers were smooth and bead-free with a common feature of porous and fibrous structures. The diameters of these blank nanofibers were mostly in the range of 240−320 nm, and the average diameters were 249.76 ± 38.02 nm. When embedded with AgNPs, there was no obvious change for the microstructure of nanofibers, although the average diameters gradually decreased with increase in the addition of AgNPs. The trend of decreasing diameters could be attributed to the increased conductivity when inducing AgNPs. For a higher conductivity, jet generated by spinning solutions became less resistant to the stretching repulsive forces of charge during the electrospinning process, resulting in a decrease in the diameter of AgNPs-embedded nanofibers. The relationships between the “controllable” parameters (e.g., spinning solution conductivity) and the resultant nanofiber diameter are difficult to assess in an accurate manner. While it was reported that electrospinning characteristics (e.g., Taylor cone’s angle) that strongly depend of a series of “controllable” parameters are highly correlated with the resultant nanofiber diameter, suggesting that they have the potential applications for accurate predictions of nanofiber size [35].

**Figure 3:**
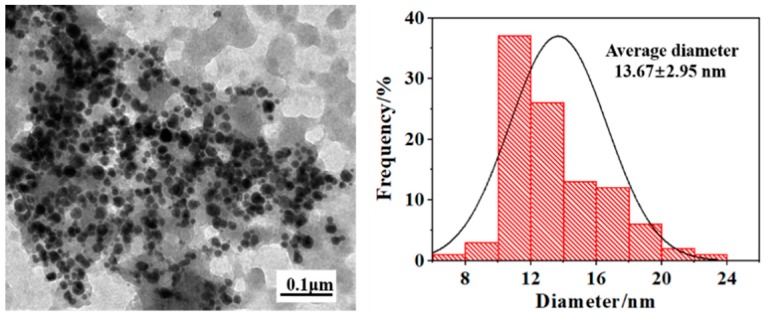
TEM images and diameter distributions of the as-synthesized AgNPs.

**Figure 4:**
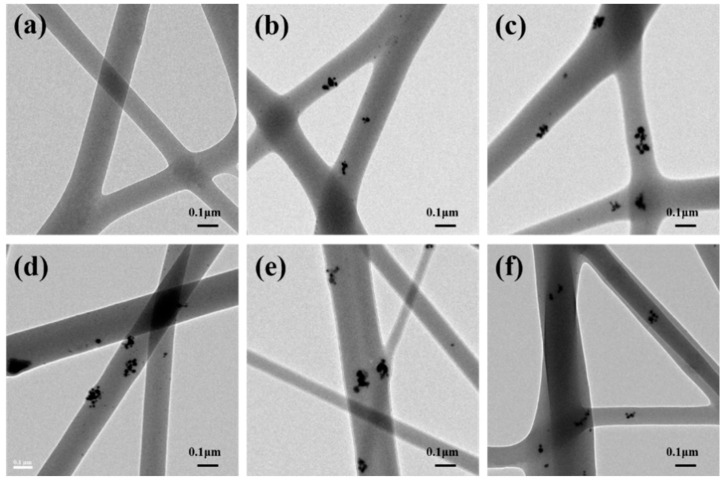
TEM images of FK/PVA/PEO composite nanofibers: (**a**) 0%-AgNPs, (**b**) 0.6%-AgNPs, (**c**) 1.2%-AgNPs, (**d**) 1.8%-AgNPs, (**e**) 2.4%-AgNPs, (**f**) 3.0%-AgNPs.

The morphology and size of the as-synthesized AgNPs was characterized using TEM. It is showed in Figure 3 that AgNPs exhibit sphere shape with an average diameter of 13.67 nm. In order to get a better insight in the dispersion of AgNPs in the composite nanofibers, TEM observations of the AgNPs-induced FK/PVA/PEO nanofibers were performed and the results are shown in Figure 4. Figure 4a shows that the composite nanofibers had a homogeneous microstructure and no distinct phase separation, confirming the compatibility and miscibility of three components in the composite nanofibers. As shown in Figure 4b–f, AgNPs were clearly identifiable in AgNPs-induced samples, which indicated that AgNPs were successfully embedded inside FK/PVA/PEO nanofibers. It is also found that AgNPs did not disperse well in the nanofibers and aggregation phenomenon is observed. This is probably because AgNPs are easy to move in the jet during the electrospinning process, because of their high conductivity. Compared with the SEM images, the amount of nanofibers observed in TEM images was quite less because of the very short collecting time on a copper grid.

### 3.2. EDX Analysis

**Figure 5:**
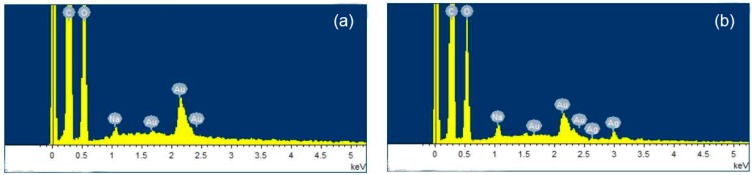
Energy-dispersive X-ray spectra FK/PVA/PEO composite nanofibers: (**a**) 0%-AgNPs, (**b**) 3%-AgNPs.

**Figure 6:**
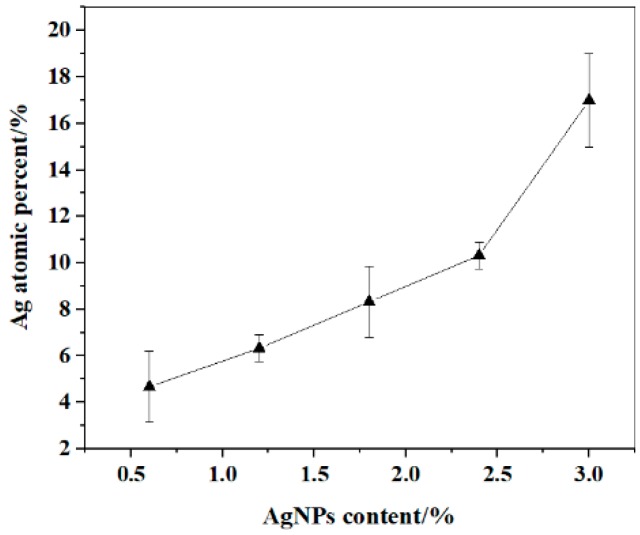
EDX measured variation curve of Ag relative atomic percentage on the surface part of the nanofibers with different AgNPs content.

EDX was used to analyze the elemental constitution of composite nanofibers. The results are shown in Figure 5 and Figure 6. Figure 5a shows that the FK/PVA/PEO composite nanofibers (0%-AgNPs) were primarily composed of C, O, Na, and Au elements. The existence of Na came from the NaOH solution when dissolving the FK powders for preparing electrospinning solutions. The existence of Au element was because of the Au coated on the surface of the samples before SEM analysis to increase the conductivity of samples. Although Figure 5b shows a new peak at 3 keV in AgNPs incorporated nanofibers (3%-AgNPs), which was attributed to Ag. Furthermore, Figure 6 shows that the Ag relative atomic percentage measured on the surface part of AgNPs-induced nanofibers increased with the increase in AgNPs content. Therefore, EDX analysis further confirmed that AgNPs were embedded in the FK/PVA/PEO composite nanofibers.

### 3.3. XRD Analysis

**Figure 7:**
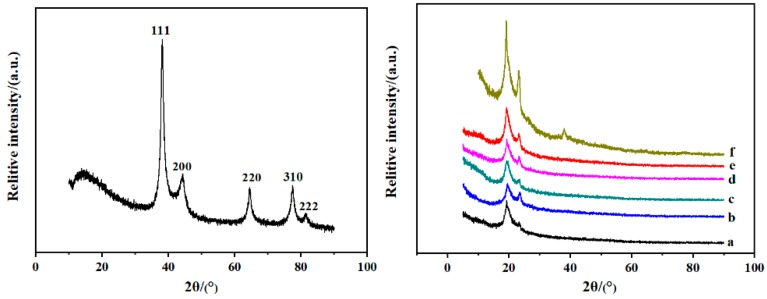
XRD patterns of the as-synthesized AgNPs (**left**) and FK/PVA/PEO composite nanofibers (**right**): (**a**) 0%-AgNPs, (**b**) 0.6%-AgNPs, (**c**) 1.2%-AgNPs, (**d**) 1.8%-AgNPs, (**e**) 2.4%-AgNPs, (**f**) 3%-AgNPs.

Figure 7 shows the XRD patterns of the as-synthesized AgNPs and FK/PVA/PEO composite nanofibers. It is apparent that AgNPs exhibits multiple diffraction peaks at 2θ of 38.2°, 44.3°, 64.5°, 77.5°, and 81.6°, which are attributed to diffractions from the (111), (200), (220), (311), and (222) lattice planes of face-centered cubic (fcc) of silver. With respect to the composite nanofibers, the diffraction peak located at 2θ of 19° is attributed to the characteristic diffraction peak of PVA, PEO, and FK [36,37]. The diffraction peak at 2θ of 23° is due to the crystal structure of PEO [36]. Compared with the AgNPs-free sample (a), the diffraction peak intensity of AgNPs-embedded samples (b–f) at 2θ of 19° and 23° becomes stronger. Moreover, when the amount of AgNPs increased to 3%, nanofibers (f) exhibits an extra diffraction peak at 2θ of 38.2°, which is attributed to diffraction from the (111) lattice plane of AgNPs. Thus, it is indicated that the incorporation of AgNPs is favorable to the crystallization of the composite nanofibers.

### 3.4. TG Analysis

**Figure 8:**
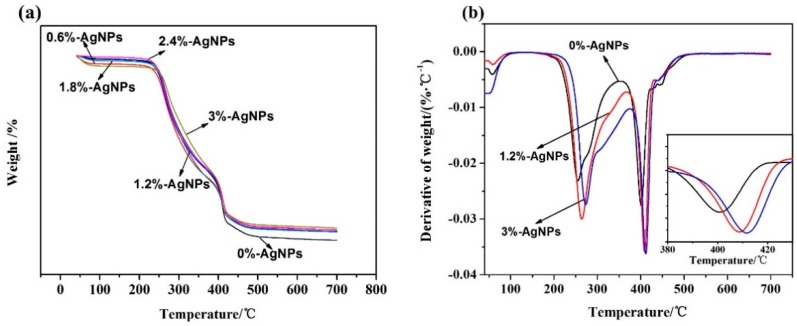
TG (**a**) and DTG (**b**) curves of FK/PVA/PEO composite nanofibers.

Figure 8a shows the TG curves of AgNPs-embedded FK/PVA/PEO nanofibers. The weight loss process of composite nanofibers could be divided into three stages. The first weight loss below 100 °C was attributed to evaporating the adsorbed moisture, which revealed the excellent hydrophilicity of nanofibers. The second weight loss stage was 225–350 °C, which was determined by the decomposition of amino acid residues of FK, the breakage of peptide bonds, and the thermal degradation of PVA [38]. In the third stage, the weight loss occurred at 350–450 °C, corresponding to the thermal decomposition of PEO and composite nanofibers. DTG analysis was used to obtain a better insight in the differences of thermal stability with different AgNPs contents. DTG curves are so close that the selected curves of 0%-AgNPs, 1.2%-AgNPs, and 3%-AgNPs can be observed in Figure 8b. Table 2 lists the TGA data of all curves. T_d_, T_max1_, and T_max2_ significantly increased with increased content of AgNPs in the composite nanofibers. The increased thermal stability of composite nanofibers was obviously because of embedded AgNPs, which is a type of metal and has a boiling point at 2162 °C [39]. Furthermore, AgNPs reduced the mobility of polymer chains, which suppressed the transfer of free radicals, thus resulting in inhibiting the inter-chain reaction. Therefore, the decomposition of AgNPs-embedded composite nanofibers occurred at higher temperatures [40]. It was also possibly because that AgNPs interacted with degradation volatiles, which thus delayed their diffusion from the nanofibers matrix [41].

### 3.5. Tensile Properties

Table 3 shows the TS and EAB of FK/PVA/PEO composite nanofibers prepared with different contents of AgNPs. Note that 0%-AgNPs exhibited an average TS value of 2.62 MPa and an average EAB value of 42.75%. The TS of the composite nanofibers initially increased, and then decreased with the increased content of AgNPs, whereas 1.2%-AgNPs showed the highest average TS value of 5.52 MPa. Although 1.2%-AgNPs reached the highest level of the average EAB value (51.50%), it could be concluded that the tensile properties of FK/PVA/PEO composite nanofibers were significantly improved when embedded with AgNPs. Note that the most appropriate AgNPs content was 1.2%, and similar results were observed in chitosan/PEO nanofibers mats by Wang [42]. It was reported that the nanofiber composite containing 2% AgNO_3_ had the highest TS (7.54 MPa) with the largest EAB (27.47%), and the enhancement in mechanical properties was possibly related to the excellent crystallinity of AgNPs by the appropriate increment of AgNO_3_ content. As shown in Figure 7, the crystallinity of the FK/PVA/PEO nanofibers increased when embedded with AgNPs. Thus, the mechanical properties of composite nanofibers enhanced by appropriate content of AgNPs. Furthermore, in our work, when AgNPs content was ≤1.2%, AgNPs could be dispersed into the composite nanofibers matrix, and the interfacial interaction between nanofibers matrix and AgNPs enhanced with increase in the content of AgNPs. Thus, it could transfer load or force from the nanofibers matrix to AgNPs filler to a certain degree during tensile deformation. However, the decrease in tensile properties when AgNPs content exceeded 1.2% was probably because of the destruction of regularity and compactness of nanofibers affected by aggregating the superabundant AgNPs. While Maharjan [43] reported that AgNPs slightly decreased the mechanical properties of PU-Zein (2:1) composite fibers, the incorporation of AgNPs might have hindered the proper orientation of the polymer molecules during solidification.

### 3.6. Antibacterial Activities

**Figure 9:**
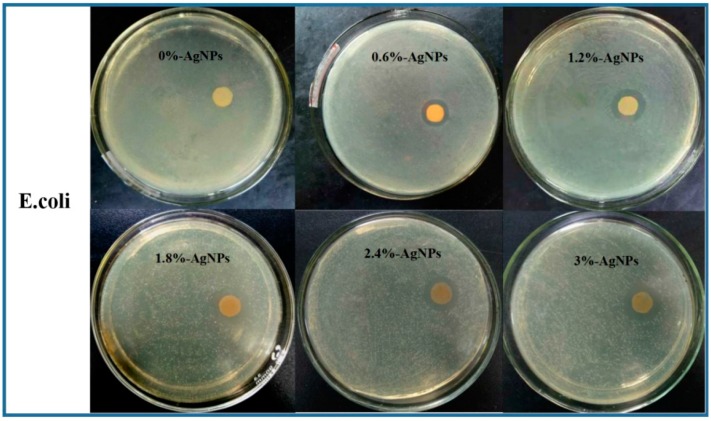

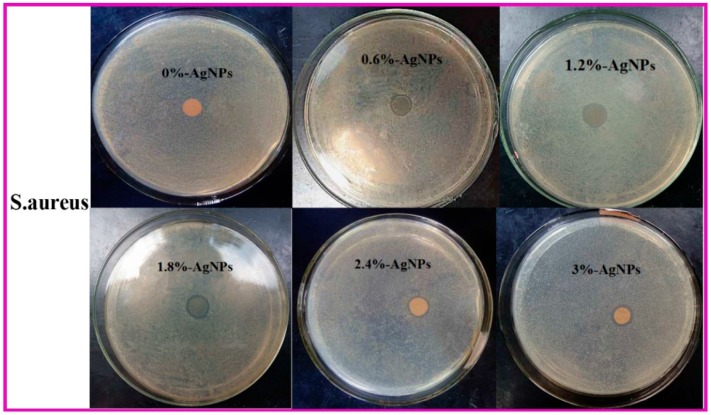
The antibacterial inhibition zone of composite nanofibers against *E. coli* and *S. aureus.*

The antibacterial activities of AgNPs embedded composite nanofibers were evaluated using the inhibition zone method. As shown in Figure 9 and Table 4, there were no inhibition zones exhibited around the 0%-AgNPs disc samples in both bacteria agar plates as expected, indicating that the FK/PVA/PEO composite nanofibers without AgNPs had no antibacterial activity. While incorporating the AgNPs caused antibacterial activity, the nanofibers showed significant inhibition against *E. coli* and *S. aureus*. Obviously, the antibacterial effect of composite nanofibers was attributed to AgNPs. The proposed mechanism is that AgNPs accumulated in the bacterial cell wall, resulting in an increase in the permeability of cell membranes [1,44]. Another mechanism suggests that AgNPs reacted with the thiol groups (–SH) of cysteine and phosphorus compounds on the cell wall and disturbs the respiration and replication processes, thereby causing cell death [45,46].

Gram-negative bacteria are more difficult to be killed than Gram-positive bacteria because they possess denser cell wall structures, which make them much less permeable to most antibacterial agents. Thus, many nanofibers incorporated with AgNPs were reported to show greater growth-inhibiting efficacy against Gram-positive *S. aureus* than Gram-negative *E. coli* [47,48,49,50]. Nevertheless, clearer and larger diameter of the inhibition zone against *E. coli* (Gram-negative) was observed compared to *S. aureus* (Gram-positive) in this work, indicating that AgNPs-embedded FK/PVA/PEO composite nanofibers have better antibacterial activity against *E. coli* than *S. aureus*. Similar to our results, a higher antimicrobial activity against *E. coli* has been reported for AgNPs-incorporated silk fibroin nanofibrous mats and polycaprolactone–chitosan coaxial nanofibers [1,51].

## 4. Conclusions

Herein, AgNPs-embedded FK/PVA/PEO antibacterial composite nanofibers were successfully prepared using an electrospinning process. AgNPs were clearly identifiable in TEM images. Furthermore, the Ag element existed in EDX spectra, and the Ag relative atomic percentage in the composite nanofibers increased with increase in AgNPs content in the spinning solution. These confirmed the embedding of AgNPs in the FK/PVA/PEO nanofibers. Morphology observation by SEM showed that AgNPs-embedded composite nanofibers were smooth and bead-free. Compared with blank samples, the average diameters of AgNPs incorporated nanofibers gradually decreased with increasing amount of AgNPs. The crystallinities, thermal stabilities, and antibacterial activities of composite nanofibers were reinforced when embedded with AgNPs. Both the TS and EAB reached the highest level at an AgNPs content of 1.2%. Antibacterial assays showed that the AgNPs-embedded FK/PVA/PEO nanofibers exhibited antibacterial activities against both Gram-positive (*S. aureus*) and Gram-negative (*E. coli*) bacteria. Clearer and larger circular inhibition zone was observed against *E. coli* compared with *S. aureus*. In summary, the AgNPs-embedded and FK-incorporated composite nanofibers exhibited the potential for a broad range of applications in the biomaterial field.

## Figures and Tables

**Table 1:** Average diameters of FK/PVA/PEO composite nanofibers with different AgNPs contents.

Samples	Average Diameters (nm)
0%-AgNPs	249.76 ± 38.02
0.6%-AgNPs	209.41 ± 21.93
1.2%-AgNPs	187.76 ± 27.90
1.8%-AgNPs	179.55 ± 27.95
2.4%-AgNPs	167.78 ± 23.38
3.0%-AgNPs	160.30 ± 25.53

**Table 2:** TGA data of FK/PVA/PEO composite nanofibers with different AgNPs amounts.

Sample (%)	T_10_ (°C)	T_max1_ (°C)	T_max2_ (°C)
0%-AgNPs	240	255	406
0.6%-AgNPs	245	262	408
1.2%-AgNPs	250	264	409
1.8%-AgNPs	253	267	410
2.4%-AgNPs	254	272	411
3%-AgNPs	256	273	413

Note: T_10_ is the temperature of 10% mass loss. T_max1_ and T_max2_ are the temperature with the fastest mass loss in the second and third weight loss stage, respectively.

**Table 3:** Tensile strength (TS) and elongation at break (EAB) of FK/PVA/PEO composite nanofibers with different AgNPs amounts.

Samples	TS (MPa)	EAB (%)
0%-AgNPs	2.62 ± 0.51	42.75 ± 0.25
0.6%-AgNPs	4.30 ± 0.64	31.25 ± 5.30
1.2%-AgNPs	5.52 ± 0.71	51.50 ± 9.19
1.8%-AgNPs	4.83 ± 1.23	30.50 ± 5.57
2.4%-AgNPs	2.60 ± 0.51	20.93 ± 10.42
3.0%-AgNPs	2.38 ± 0.57	12.5 ± 3.27

**Table 4:** The diameter of antibacterial inhibition zone of the FK/PVA/PEO composite nanofibers against *E. coli* and *S. aureus*.

Samples	Diameter of Antibacterial Inhibition (mm)	
	*E. coli*	*S. aureus*
0%-AgNPs	0	0
0.6%-AgNPs	5.02	2.12
1.2%-AgNPs	8.24	2.08
1.8%-AgNPs	8.40	1.70
2.4%-AgNPs	8.17	1.56
3.0%-AgNPs	8.60	2.96
